# Supplementary material for: Are Further Interventions Needed to Prevent and Manage Hospital-Acquired Hyponatraemia? A Nationwide Cross-Sectional Survey of IV Fluid Prescribing Practices
Source: J Clin Med. 2020 Aug 29;9(9):2790. doi: 10.3390/jcm9092790 (PMC7565867; doi:10.3390/jcm9092790)
Supplement: Supplementary file 1 [file jcm-09-02790-s001.pdf]

Journal of Clinical Medicine

MDPI

Are Further Interventions Needed to Prevent and Manage Hospital-Acquired Hyponatraemia? A Nationwide Cross-sectional Survey of IV Fluid Prescribing Practices

Per Sindahl [1,2,3,*], Christian Overgaard-Steensen [4], Helle Wallach-Kildemoes [5], Marie Louise De Bruin [3], Hubert GM Leufkens [2], Kaare Kemp [1] and Helga Gardarsdottir [2,6]

Questionnaire about intravenous fluids and hyponatraemia

[A cross (X) is used to indicate the correct answer. References was not part of the questionnaire presented for the participants]

Background questions

1. What is your gender?

   ☐ Female

   ☐ Male

   ☐ Other

---

2. Which of the following categories describe your age? (check only one)

   ☐ 18 – 24 years

   ☐ 25 – 34 years

   ☐ 35 – 44 years

   ☐ 45 – 54 years

   ☐ 55 – 64 years

   ☐ 65 years or older

---

3. How many patients do you treat with IV fluids in an average week? (check only one)

☐ 0 patients

☐ 1 - 5 patients

☐ 6 - 10 patients

☐ 11 - 15 patients

☐ More than 15 patients

---

4. What is your current position? (check only one)

Physician:

☐ Foundation doctor year 1 (FY1)

☐ Foundation doctor year 2 (FY2)

☐ Specialty registrar

☐ Consultant

☐ Other, please specify:____

---

5. How many years have you been practising as a physician? (check only one)

☐ 0 – 5 years

☐ 6 – 10 years

☐ 11 – 15 years

☐ 16 – 20 years

☐ 21 – 25 years

☐ 26 years or more

Please note that it is not allowed to go back and rectify previous questions.
Four scenarios now follow, illustrating typical clinical situations in the emergency department.

Scenario 1
An otherwise healthy 18-year-old girl is hospitalised on suspicion of meningitis. She has thrown up and has diarrhea. On examination, she appears pale with cold skin, a slightly increased heart rate, normal blood pressure, and with decreased level of consciousness (Glasgow Coma Scale [GSC] – score 14). Laboratory tests show:

- pH 7.4 (ref.: 7.35 – 7.45);
- Plasma (P)-glukose 5.0 mmol/L (ref.: 4.2 – 6.3 mmol/l);
- P-[Na^+] 138 mmol/L (ref.: 137-145 mmol/L);
- P-[K^+] 3.6 mmol/L (ref.: 3.5 – 4.4 mmol/L).

The patient is unable to take fluids per os.

6. Which of the following is your first-line treatment of choice for this patient? (check only one)

☐ **Darrow-glucose**
(1 L contains 31 mmol sodium [1 g sodium chloride and 1.56 g sodium lactate], 55 g glucose monohydrate (278 mmol glucose), 0.67 g potassium chloride)

X **Isotonic saline**[1]
(1 L contains 9 g [154 mmol] sodium chloride)

☐ **Ringer's acetate**
(1 L contains 130 mmol sodium [4.1 g sodium acetate and 5.9 g sodium chloride], 0.295 g calcium chloride, 0.3 g potassium chloride, 0.2 g magnesium chloride)

☐ **3% NaCl**
(1 L contains 30 g [513 mmol] sodium chloride)

☐ **Potassium-sodium-glucose**
(1 L contains 2.338 g sodium chloride [40 mmol], 55 g [278 mmol] glucose [as monohydrate], and 1.492 g potassium chloride)

☐ **0.9% NaCl with 5% glucose**
(1 L contains 50 g [278 mmol] glucose and 9 g [154 mmol] sodium chloride)

☐ **0.45% sodium chloride with 2.5% glucose isotonic**
(1 L contains 25 g [139 mmol] water free glucose and 4.5 g sodium chloride [77 mmol])

☐ **Glucose 5% isotonic**
(1 liter contains 55 g (278 mmol) glucose)

☐ **Do not know**

---

[1] Isotonic saline is recommended in patients with increased intracranial pressure [1–3].

Scenario 2

A 5-year old boy arrives at the emergency department with a head injury after falling from a bike. He has headache and nausea, but no vomiting or signs of hypovolaemia. He has been unconscious for half an hour, however, the CT scan, clinical examination and laboratory results are all normal.

- pH 7,4 (ref.: 7.35 – 7.45);
- Plasma (P)-glukose 5.8 mmol/L (ref.: 4.2 – 6.3 mmol/l);
- P-[Na^+] 138 mmol/L (ref.: 137-145 mmol/L);
- P-[K^+] 3.6 mmol/L (ref.: 3.5 – 4.4 mmol/L).

Due to nausea, the patient is unable to take fluids per os.

7. Which of the following is your first-line treatment of choice for this patient? (check only one)

☐ **Potassium-sodium-glucose**
(1 L contains 2.338 g sodium chloride [40 mmol], 55 g [278 mmol] glucose [as monohydrate], and 1.492 g potassium chloride)

☐ **3% NaCl**
(1 L contains 30 g [513 mmol] sodium chloride)

☐ **Ringer's acetate**
(1 L contains 130 mmol sodium [4.1 g sodium acetate and 5.9 g sodium chloride], 0.295 g calcium chloride, 0.3 g potassium chloride, 0.2 g magnesium chloride)

☐ **0.45% sodium chloride with 2.5% glucose isotonic**
(1 L contains 25 g [139 mmol] water free glucose and 4.5 g sodium chloride [77 mmol])

☐ **Darrow-glucose**
(1 L contains 31 mmol sodium [1 g sodium chloride and 1.56 g sodium lactate], 55 g glucose monohydrate (278 mmol glucose), 0.67 g potassium chloride)

☐ **Isotonic saline**
(1 L contains 9 g [154 mmol] sodium chloride)

X **0.9% NaCl with 5% glucose**[2]
(1 L contains 50 g [278 mmol] glucose and 9 g [154 mmol] sodium chloride)

☐ **Glucose 5% isotonic**
(1 liter contains 55 g (278 mmol) glucose)

☐ **Do not know**

---

[2] Isotonic saline with glucose is recommended for maintenance treatment in children [4,5].

Scenario 3

A 75-year-old woman arrives at the emergency department with hip fracture after a fall. There are no signs of head injury. The patient has had a poor appetite for a long time. Medical history includes thiazide diuretics for hypertension, but otherwise she is healthy. Clinical examination shows symptoms of hypovolaemia: cold and pale skin, heart rate at 100 bpm, and a slightly increased respiratory rate. Laboratory findings:

- pH 7.4 (ref.: 7.35 – 7.45);
- Plasma (P)-glukose 6 mmol/L (ref.: 4.2 – 6.3 mmol/l);
- P-[Na^+] 110 mmol/L (ref.: 137 - 145 mmol/L);
- P-[K^+] 3.6 mmol/L (ref.: 3.5 – 4.4 mmol/L).

8. Which of the following is your first-line treatment of choice for this patient? (check only one)

☐ **Darrow-glucose**
(1 L contains 31 mmol sodium [1 g sodium chloride and 1.56 g sodium lactate], 55 g glucose monohydrate (278 mmol glucose), 0.67 g potassium chloride)

X **Isotonic saline**[3]
(1 L contains 9 g [154 mmol] sodium chloride)

☐ **3% NaCl**
(1 L contains 30 g [513 mmol] sodium chloride)

☐ **Potassium-sodium-glucose**
(1 L contains 2.338 g sodium chloride [40 mmol], 55 g [278 mmol] glucose [as monohydrate], and 1.492 g potassium chloride)

☐ **0.9% NaCl with 5% glucose**
(1 L contains 50 g [278 mmol] glucose and 9 g [154 mmol] sodium chloride)

☐ **0.45% sodium chloride with 2.5% glucose isotonic**
(1 L contains 25 g [139 mmol] water free glucose and 4.5 g sodium chloride [77 mmol])

☐ **Glucose 5% isotonic**
(1 liter contains 55 g (278 mmol) glucose)

X **Ringer's acetate**
(1 L contains 130 mmol sodium [4.1 g sodium acetate and 5.9 g sodium chloride], 0.295 g calcium chloride, 0.3 g potassium chloride, 0.2 g magnesium chloride)

☐ **Do not know**

---

[3] Either isotonic saline or Ringers is recommended in hypovolaemic patients with asymptomatic hyponatraemia [1,6].

Scenario 4

A 28-year old woman is hospitalised on suspicion of medication poisoning and large intake of water. She vomits and complains about headaches. She exhibits strange behavior, has muscle rigidity and a Glasgow Coma Scale (GSC) score of 14. ABC is normal. Arterial blood gas shows:

- pH 7.48 (ref.: 7.35 – 7.45);
- Plasma (P)-glukose 5.4 mmol/L (ref.: 4.2 – 6.3 mmol/l);
- P-[Na^+] 118 mmol/L (ref.: 137 - 145 mmol/L);
- P-[K^+] 3.6 mmol/L (ref.: 3.5 – 4.4 mmol/L);
- pO_2 14 kPa (ref.: 11.1 – 14.4 kPa);
- pCO_2 4 kPa (ref.: 4.3 – 5.7 kPa).

9. Which of the following is your first-line treatment of choice for this patient? (check only one)

☐ **Potassium-sodium-glucose**
(1 L contains 2.338 g sodium chloride [40 mmol], 55 g [278 mmol] glucose [as monohydrate], and 1.492 g potassium chloride)

☐ **Isotonic saline**
(1 L contains 9 g [154 mmol] sodium chloride)

☐ **Ringer's acetate**
(1 L contains 130 mmol sodium [4.1 g sodium acetate and 5.9 g sodium chloride], 0.295 g calcium chloride, 0.3 g potassium chloride, 0.2 g magnesium chloride)

X **3% NaCl**[4]
(1 L contains 30 g [513 mmol] sodium chloride)

☐ **0.9% NaCl with 5% glucose**
(1 L contains 50 g [278 mmol] glucose and 9 g [154 mmol] sodium chloride)

☐ **Darrow-glucose**
(1 L contains 31 mmol sodium [1 g sodium chloride and 1.56 g sodium lactate], 55 g glucose monohydrate (278 mmol glucose), 0.67 g potassium chloride)

☐ **0.45% sodium chloride with 2.5% glucose isotonic**
(1 L contains 25 g [139 mmol] water free glucose and 4.5 g sodium chloride [77 mmol])

☐ **Glucose 5% isotonic**
(1 liter contains 55 g (278 mmol) glucose)

☐ **Do not know**

---

[4] Hypertonic (3%) saline is recommended for patients with symptomatic hyponatraemia (e.g. altered level of consciousness (DK), vomiting (EU), polydipsia (US), headache (UpToDate)) [1,7–9].

Renal water excretion

Imagine an acutely ill patient in need of IV fluid.

10. Which of the following sentences are correct? (check only one)

☐ Most often acutely ill patients in need of IV fluids have increased renal water excretion

X[5] Most often acutely ill patients in need of IV fluids have decreased renal water excretion

☐ Most often acutely ill patients in need of IV fluids have normal renal water excretion

☐ Do not know

---

[5] Renal water excretion is often reduced in acutely ill patients [10].

IV fluids impact on plasma sodium concentration (P-Na)

Four questions now follow for which you should imagine a patient at risk of reduced renal water excretion, and in need of a larger amount (> 20 ml / kg) of different IV fluids.

11. What is the most likely way that the following IV fluids will affect the plasma sodium concentration (P-Na) in such a patient?

    Please tick one box only for each IV fluid

	Large increase in P-Na with a risk of sodium overload	Slight increase in P-Na	Un-changed	Slight decrease in P-Na	Large decrease in P-Na with a risk of hypo-natriaemia	Do not know
**Ringer's lactate**[6] (1 L contains 130 mmol sodium)				X		
**Darrow-glucose** (1 L contains 31 mmol sodium)					X	
**Potassium-sodium-glucose** (1 L contains 40 mmol)					X	
**0.9% NaCl and 5% glucose** (1 L contains 154 mmol sodium chloride)			X			

[6] Ringer's lactate is hypotonic because it have a lower sodium concentration than extracellular fluid [1,11].

Hyperglycaemia and plasma sodium

12. Which one of the following sentences are correct? (check only one)

X	In case of increased blood sugar (above 12 mmol/L), the measured plasma sodium (P-Na) must be corrected because the measured P-Na is "falsely low".[7]
☐	In case of increased blood sugar (above 12 mmol/L), the measured plasma sodium (P-Na) must be corrected because the measured P-Na is "falsely high".
☐	In case of increased blood sugar (above 12 mmol/L), there is no reason to correct the measured plasma sodium (P-Na) because the measured P-Na is correct.
☐	Do not know.

Patients at high risk of severe symptoms upon inappropriate IV fluid treatment

13. Which of these diseases/symptoms may be indicative of potentially increased intracranial pressure (ICP) and therefore require special attention in relation to fluid treatment since even minor changes in plasma sodium may be critical? (check only one for each symptom/disease)

	Yes	No	Do not know
**Meningitis[8]**	x		
	Yes	No	Do not know
**Shortness of breath**		x	
	Yes	No	Do not know
**Concussion**	x		
	Yes	No	Do not know
**Chest pain**		x	
	Yes	No	Do not know
**Seizure**	x		
	Yes	No	Do not know
**Acute liver failure**	x		
	Yes	No	Do not know
**Acute abdomen**		x	
	Yes	No	Do not know
**Hip fracture**		x	

---

7 The measured plasma sodium is falsely low and must be corrected in the presence of hyperglycaemia [1,7].

8 Meningitis, concussion, seizure and acute liver failure are all causes of increased intracranial pressure [2].

Severe symptoms of hyponatraemia

14. Which of the following symptoms are indicative of severe symptoms of hyponatraemia and require acute treatment of hyponatraemia? (check only one for each symptom)

	Yes	No	Do not know
**Altered level of consciousness** [9]	x		
	Yes	No	Do not know
**Seizure**	x		
	Yes	No	Do not know
**Infection**		x	
	Yes	No	Do not know
**Chest pain**		x	
	Yes	No	Do not know
**Muscle rigidity**	x		
	Yes	No	Do not know
**Anaemia**		x	

[9] Examples of severe symptoms of hyponatraemia vary across guidelines. According to the Danish Endocrine Society altered level of consciousness, seizures and muscle rigidity are severe symptoms of hyponatraemia [1].

Prevention and treatment of overcorrection

Imagine a patient at high risk of brain damage (e.g. P-Na <115, PK < 2.5, malnutrition and/or severe liver disease) due to osmotic demyelination.

15. a) What is the maximum correction of plasma sodium per day for such a patient? (check only one)

X 6 mmol/l[10]

☐ 8 mmol/l

☐ 16 mmol/l

☐ 20 mmol/l

☐ Do not know

Imagine a patient without a high risk of brain damage due to osmotic demyelination.

15. b) What is the maximum correction of plasma sodium per day for such a patient? (check only one)

☐ 6 mmol/l

X 8 mmol/l

☐ 16 mmol/l

☐ 20 mmol/l

☐ Do not know

---

[10] The recommended limits for P-Na correction vary across guidelines. The Danish Endocrine Society and UpToDate recommend 6-8 mmol/L depending on the patient's symptoms [1,12].

In relation to IV fluid treatment for hyponatraemia, there is a risk that plasma sodium levels rise too rapidly and exceed the maximum recommended limit of plasma sodium.

16. How would you prevent plasma sodium from rising too rapidly in relation to IV fluid treatment of hyponatraemia, and thus exceeding the recommended limits for plasma sodium (P-Na) correction? (select all that apply)

X I record the administration of IV fluids

☐ I recommend fluid restriction

X I record the first low P-Na level

X I monitor P-Na regularly

☐ I administer 5% glucose

X I record the maximum recommended increase of P-Na

☐ I encourage the patient to drink water

☐ Do not know

---

17. Which of the following would you administer to a patient whose plasma sodium concentration raise too rapidly? (select all that apply)

X Water per os[11]

☐ Fluid restriction (fluid intake is limited to less than 1 liter/day)

☐ Isotonic saline (1 L contains 9 g [154 mmol] sodium chloride)

☐ Ringer's acetate (1 L contains 130 mmol sodium [4.1 g sodium acetate and 5.9 g sodium chloride], 0.295 g calcium chloride, 0.3 g potassium chloride, 0.2 g magnesium chloride)

☐ 3% NaCl (1 L contains 30 g [513 mmol] sodium chloride)

X Glucose 5% isotonic (1 liter contains 55 g [278 mmol] glucose)

☐ Do not know

---

[11] Water per os or 5% glucose is recommended for patients in whom plasma sodium concentration rises too rapidly [1,7,8,12].

18. What is the most common cause of over-correction? (check only one)

X Large diuresis[12]

☐ Increased sodium secretion

☐ Increased water intake

☐ Inadequate water intake

☐ Increased sodium intake

☐ Reduced renal water excretion

[12] Large diuresis is recognised as a common cause of over-correction in the guidelines set by the Danish Endocrine Society and Sterns et al. [1,13].

Final questions

19. Did you use any aids to complete the questionnaire?

☐ No

☐ Yes

If yes, which did you use? (select all that apply)

☐ The treatment guideline of the department

☐ Pro.medicin.dk

☐ The guideline on hyponatraemia from the Association of Clinical Endocrinologists

☐ Other:____

Thank you very much for your participation. If you have any comments to the questionnaire or research project, you can write them below or send an email to Per Sindahl, pesi@dkma.dk:

References

1. Dansk Endokrinologisk Selskab NBV Endokrinologi: Hyponatriæmi [National Treatment Guideline Endocrinology: Hyponatraemia] Available online: http://www.endocrinology.dk/index.php/6-andre-endokrinologiske-sygdomme/2-hyponatriaemi (accessed on Sep 23, 2019).
2. Evaluation and management of elevated intracranial pressure in adults - UpToDate Available online: https://www.uptodate.com/contents/evaluation-and-management-of-elevated-intracranial-pressure-in-adults?search=icp%20management&source=search_result&selectedTitle=1~150&usage_type=default&display_rank=1 (accessed on Apr 5, 2020).
3. Pharmacovigilance Risk Assessment Committee Recommendations on signals adopted at the 3-6 July 2017 PRAC Available online: https://www.ema.europa.eu/en/committees/prac/prac-agendas-minutes-highlights#minutes-section.
4. Feld, L.G.; Neuspiel, D.R.; Foster, B.A.; Leu, M.G.; Garber, M.D.; Austin, K.; Basu, R.K.; Conway, E.E.; Fehr, J.J.; Hawkins, C.; et al. Clinical Practice Guideline: Maintenance Intravenous Fluids in Children. *Pediatrics* **2018**, *142*, doi:10.1542/peds.2018-3083.
5. Maintenance intravenous fluid therapy in children - UpToDate Available online: https://www.uptodate.com/contents/maintenance-intravenous-fluid-therapy-in-children?search=maintenance%20fluids%20children&source=search_result&selectedTitle=1~150&usage_type=default&display_rank=1 (accessed on Aug 12, 2020).
6. Liamis, G.; Filippatos, T.D.; Elisaf, M.S. Correction of hypovolemia with crystalloid fluids: Individualizing infusion therapy. *Postgrad. Med.* **2015**, *127*, 405–412, doi:10.1080/00325481.2015.1029421.
7. Spasovski, G.; Vanholder, R.; Allolio, B.; Annane, D.; Ball, S.; Bichet, D.; Decaux, G.; Fenske, W.; Hoorn, E.J.; Hoorn, E.; et al. Clinical practice guideline on diagnosis and treatment of hyponatraemia. *Intensive Care Med.* **2014**, *40*, 320–331, doi:10.1007/s00134-014-3210-2.
8. Verbalis, J.G.; Goldsmith, S.R.; Greenberg, A.; Korzelius, C.; Schrier, R.W.; Sterns, R.H.; Thompson, C.J. Diagnosis, Evaluation, and Treatment of Hyponatremia: Expert Panel Recommendations. *Am. J. Med.* **2013**, *126*, S1–S42, doi:10.1016/j.amjmed.2013.07.006.
9. Overview of the treatment of hyponatremia in adults - UpToDate Available online: https://www.uptodate.com/contents/overview-of-the-treatment-of-hyponatremia-in-adults?search=hyponatremia&source=search_result&selectedTitle=1~150&usage_type=default&display_rank=1#H2818747047 (accessed on Aug 11, 2020).
10. Moritz, M.L.; Ayus, J.C. Maintenance Intravenous Fluids in Acutely Ill Patients. *N. Engl. J. Med.* **2015**, *373*, 1350–1360, doi:10.1056/NEJMra1412877.
11. Myburgh, J.A.; Mythen, M.G. Resuscitation Fluids. *N. Engl. J. Med.* **2013**, *369*, 1243–1251, doi:10.1056/NEJMra1208627.
12. Osmotic demyelination syndrome (ODS) and overly rapid correction of hyponatremia - UpToDate Available online: https://www.uptodate.com/contents/osmotic-demyelination-syndrome-ods-and-overly-rapid-correction-of-hyponatremia?search=hyponatremia%20treatment&source=search_result&selectedTitle=7~150&usage_type=default&display_rank=5 (accessed on Aug 12, 2020).
13. Sterns, R.H.; Nigwekar, S.U.; Hix, J.K. The Treatment of Hyponatremia. *Semin. Nephrol.* **2009**, *29*, 282–299, doi:10.1016/j.semnephrol.2009.03.002.
